# Feasibility of β-Sheet Breaker Peptide-H102 Treatment for Alzheimer's Disease Based on β-Amyloid Hypothesis

**DOI:** 10.1371/journal.pone.0112052

**Published:** 2014-11-05

**Authors:** Lai-xiang Lin, Xiang-yu Bo, Yuan-zhen Tan, Feng-xian Sun, Ming Song, Juan Zhao, Zhi-hong Ma, Mei Li, Kai-jun Zheng, Shu-mei Xu

**Affiliations:** 1 Department of physiology, Tianjin Medical University, Tianjin, China; 2 2011 Collaborative Innovation Center of Tianjin for Medical Epigenetics, Key Laboratory of Hormone and Development (Ministry of Health), Metabolic Diseases Hospital and Tianjin Institute of Endocrinology, Tianjin Medical University, Tianjin, China; Nathan Kline Institute and New York University School of Medicine, United States of America

## Abstract

β-amyloid hypothesis is the predominant hypothesis in the study of pathogenesis of Alzheimer's disease. This hypothesis claims that aggregation and neurotoxic effects of amyloid β (Aβ) is the common pathway in a variety of etiological factors for Alzheimer's disease. Aβ peptide derives from amyloid precursor protein (APP). β-sheet breaker peptides can directly prevent and reverse protein misfolding and aggregation in conformational disorders. Based on the stereochemical structure of Aβ1-42 and aggregation character, we had designed a series of β-sheet breaker peptides in our previous work and screened out a 10-residue peptide β-sheet breaker peptide, H102. We evaluated the effects of H102 on expression of P-tau, several associated proteins, inflammatory factors and apoptosis factors, and examined the cognitive ability of APP transgenic mice by behavioral test. This study aims to validate the β-amyloid hypothesis and provide an experimental evidence for the feasibility of H102 treatment for Alzheimer's disease.

## Introduction

Alzheimer's disease (AD) is a major progressive neurodegenerative disorder in the central nervous system, a hallmark event is the misfolding and aggregation of an otherwise normal protein [1]–[3]. Several evidences on AD have highlighted the importance of protein misfolding and amyloid formation and indicated that inhibition or dissolution of protein aggregates might be a general therapeutic strategy for these disorders [3]–[5]. The major component of amyloid plaques is β-amyloid protein (Aβ), a peptide of 39–42 amino acid residues. This peptide derives from amyloid precursor protein (APP), which arranges as a highly ordered β-sheet structure forming fibrillar aggregates of different dimensions [6]–[10]. The correlation between the diffusion of amyloid plaques in the brain and the progression of the disease remains controversial [11], [12], nevertheless, the amyloid hypothesis has been investigated as a predominant hypothesis in the study of pathogenesis of AD [9]. This hypothesis claimed that Aβ aggregation initiates the disease processes of AD, which involve the effects of acetylcholine in the nervous system, damage of synaptic plasticity, formation of free radicals, disequilibrium of intracellular calcium ion distribution, chronic inflammation, excessive phosphorylation of P-tau and other physiopathologic changes. Eventually, these factors induce cell apoptosis and produce a series of clinical symptoms, including neuron death, memory lapse, cognitive ability decrease, behavior disorders and so on. Therefore, aggregation of Aβ plays a key role and is an initial factor for the pathogenesis of AD, and Aβ-based interventional therapy becomes an important research area in treatment of AD.

Secondary structure of Aβ is composed of α-helix, β-turn and β-sheet [13]. Hydrophobic carboxyl terminal mainly consists of β-sheet while hydrophilic amino terminal mainly consists of α-helix and β-turn. Under physiological conditions, hydrophobic carboxyl terminal was hidden and hydrophilic amino terminal was exposed, and Aβ is soluble [14]. Several lines of evidence have shown that a significant proportion of Aβ aggregation is driven by hydrophobic sequences [15]–[17]. Based on those studies, β-sheet was modified aiming at inhibiting Aβ fibrillogenesis [2]. β-sheet breaker peptides have been developed as a platform concept to generate compounds to directly prevent and reverse protein misfolding and aggregation for conformational disorders [2], [3], [5]. They have been also used as therapeutic agents for the diseases characterized by amyloid fibril formation [18]. The peptides intended to treat AD are based on β-sheet disrupting elements and the self-recognition motif of Aβ, the region implicated in early misfolding and protein-protein interaction [19]. The valine, a key residue for β-sheet formation, was replaced by proline, an amino acid thermodynamically unable to fit in the β-sheet structure, and a charged residue was introduced at the C-terminal part to increase the solubility [20], [21]. Compound with such sequence has been shown to prevent amyloid formation *in vitro* and *in vivo* and to weaken amyloid neurotoxicity [20], [22].

Based on the stereochemical structure and characteristic aggregation of Aβ1-42, we designed a series of β-sheet breakers peptides, including K7, L5, H100, H101, H102, and H103. Through drug screening, we selected the best one, H102, a 10-residue peptide. H102 may work via inhibiting the interconnection of β-sheet of Aβ1-42 so as to prevent or reverse misfolding and aggregation of Aβ. In addition, we demonstrated the inhibitory action of H102 on Aβ aggregation [23], its positive effects on Aβ degradation (insulin-degrading enzyme and neprilysin) [24] and the expression of the proteins [25] of synaptophysin, PSD-95 and Shank-1, which were identified to be involved in the rehabilitation of nerve synapse [26] as well as the effects of acetylcholine in the nervous system (ChAT, AChE) and free radicals (MDA and SOD) [27].This study aims to clarify the effects of H102 on inflammatory factors, P-tau and several associated proteins, apoptosis factors and behavioral changes. We also examined the cognitive ability of APP transgenic mice as behavioral test, and conducted the Morris water maze to prove the therapeutic effect of H102 in AD.

## Materials and Methods

### Mice

APP695V717I transgenic mice (weight: 28.5–36.5 g) and C57BL/6J mice with the same background and age were obtained from the Experimental Animal Research Center, Chinese Academy of Medical Sciences & Peking Union Medical College. APP695V717I transgenic mice aged 10 months were used, which overexpressed the human APP695 with the London mutation V717I and exhibited Aβ deposits. All mice had free access to food and water, and were divided randomly into model and treatment groups. Mice were housed at a room temperature of 24±1°C and with a 12∶12 h light/dark cycle with lights on at 6:00 am. Behavioral experiments were carried out during the light period between 8:00 am and 2:00 pm. All experiments were conducted in accordance with the National Institutes of Health Guide for Care and Use of Laboratory Animals and approved by the Institutional Animal Care and Use Committee of Tianjin Medical University.

### Materials

H102 was synthesized through Fmoc solid-phase synthesis and purified by high performance liquid chromatography with a purity of 95% identified by mass spectrum (Gill Biotechnology Company, Shanghai, China). H102 was a polypeptide comprising the amino acid sequence of His-Lys-Gln-Leu-Pro-Phe-Phe-Glu-Glu-Asp(HKQLPFFEED). H102 was dissolved in saline (80 µmol/L). Anti-p-Tau, anti-GSK-3β, anti-PP-2A, anti-Bcl-2, anti-Bax and DAB kit were purchased from Biosynthesis Biotechnology Co., Ltd (Beijing, China), and protein assay kit was purchased from PIERCE Co. (USA). Total protein assay kit was purchased from Tianjin Laboratory Medicine Technology Co., Ltd (Tianjin, China).

### Lateral intracerebroventricular injection of H102 into the brains of APP transgenic mice

Mice were anesthetized with 10% chloral hydrate (300 mg/kg, i.p.) and mounted onto a stereotaxic instrument so that the frontal and parietal bones of the skull were kept parallel to the surgical platform. The surgical site was shaved and sterilized with anerdian. An incision roughly 1.5 cm in length was made along the middle line of cranium to reveal the bregma and a stainless steel guide cannula (0.8 mm) was placed in the right lateral cerebral ventricle according to the predetermined stereotaxic coordinates (lateral 1.6 mm and anteroposterior 1 mm to the bregma, and horizontal 2 mm from the dura mater). The guide cannula was fixed using two adjacent stainless steel screws and dental cementin. A dummy cannula was inserted into the guide cannula to prevent occlusion and infection. Mice were allowed a minimum of seven additional days to recover from any discomfort or weight loss before treatment or behavioral test. Then APP695V717I transgenic mice were randomized into model group and H102-treated group, and C57BL/6J mice served as control group. Three µl of 80 µmol/ml H102 saline solution was injected into the lateral ventricle of H102-treated group daily, and the injection cannula was retained for an additional 1 min before being removed. The control group and model group received a daily dose of 3 µl saline, respectively by the similar route as mentioned above.

### Behavioral test

Morris water maze was composed of a round pool (80 cm in diameter and 32 cm in height) made of stainless steel and a movable platform (24 cm in height). Clean water was poured into the pool, which was mixed with milk to keep the sight of mice out of platform, and the surface of water was 1 cm higher than the platform. The temperature of water was controlled at 25.0±1.0°C. A video monitor over the pool was connected to a computer. When the fixed time was over or the animal climbed onto the platform, the computer stopped tracing, recorded the swimming route, and automatically calculated the distance of swimming, the time for finding the platform (the water escape-latency) and the original angle (the angle between the long axes of mouse body and the line of plunging point to platform at the time of plunging into water). The platform was in the center of the third quadrant. We randomly selected an entrance point in the other quadrants to put the mouse in the water. Experimental training was carried out twice a day, 90 seconds each time at different entrance points. The mouse was placed on the platform for about 20 seconds. In order to observe the relationship between the mouse and the surrounding environment, the animal was placed to face the wall in the water in one of the quadrants. If the mouse did not find the platform within 90 seconds, it was guided to the platform for a rest of 20 seconds. If the mouse found the platform within 90 seconds, it was permitted to rest there for 20 seconds and finished the training. The experiment lasted six days. On the last day of the training, the platform was removed. The time for each mouse to pass the original platform was recorded. And the original angle of the mouse and the staying time in the quadrant of the platform were recorded as the indexes of estimation (spatial probe test).

### Treatment of brain tissues

The mice were anesthetized by celiac injection with 10% chloral hydrate (400 mg/kg) and sacrificed via decapitation. Their brains were taken out rapidly on the ice table and fixed in paraformaldehyde solution containing 30% sucrose. Tissue sections (5 µm thick) were fixed and embedded in paraffin, and made sequentially along coronal profile. They were put on clean slides treated with poly-L-lysine, baked for one hour in an oven of 60°C, and then kept in a refrigerator of 4°C for immunohistochemical (IHC) test.

### Immunohistochemical test

The IHC test was performed according to the instructions of the kit. Paraffin sections were dewaxed routinely, and incubated for 15 min with 3% H_2_O_2_ to deactivate the endogenous catalase. Then the sections were put into 0.01 mol/L citrate buffer solution (pH 6.0), heated for 5 min twice in a microwave oven with an interval of 10 min for antigen repair, cooled to room temperature, and washed with 0.1 mol/L phosphate buffered solution (PBS) for 3 times (3 min each time). Then properly diluted antibody from rabbit was added, incubated overnight at 4°C, and washed with 0.1 mol/L PBS for 3 times (5 min each time). Sections were stained by diaminobenzidine (DAB) in a lucifugal condition at room temperature, washed with water sufficiently, re-dyed, dehydrated until hyaloid was formed, mounted and observed under microscope. We replaced the antibody with PBS as negative control following the same procedure.

### Western blotting

Snap-frozen brain tissue was homogenized in lysates containing 20 mM Tris-Cl (pH 7.4) and 1 mM EDTA using an Ultrasonic Cell Crusher for 3×30 seconds and centrifuged at 12000 rpm at 4°C for 2 min. A small amount of supernatant was collected for semi-quantitative detection. Samples of an equal concentration were prepared and transferred to a fresh tube respectively. Each sample of 100 µg protein was heated at 100°C for 5 min with loading buffer containing 0.125 M Tris-HCL (pH 6.8), 20% glycerol, 4% SDS, 10% mercaptoethanol and 0.002% bromphenol blue, and then separated by sodium dodecyl sulfate-polyacrylamide gel electrophoresis (SDS-PAGE) using 10% acrylamide gels. The proteins were transferred onto polyvinylidene fluoride (PVDF) membranes. Membranes were washed for 10 min in Tris buffered saline with Tween-20 (TBST) containing 20 mM Tris-HCL (pH 7.4), 150 mM NaCl and 0.05% Tween-20, and blocked in TBST containing 5% nonfat dry milk for 2 h. Blots were incubated with corresponding primary antibodies at 4°C overnight. Membranes were washed in TBST for 3×10 min, and incubated with peroxidase-conjugated goat anti-rabbit secondary antibody for 1 h at room temperature. The bands on the membranes were scanned and analyzed using a Leica microscope with MIAS-2000 Image Analysis System and normalized to signals of β-Actin.

### Statistical analysis

Data were expressed as mean ± SEM. Repeated measures ANOVA or one-way ANOVA was applied in accordance with repeated measures design or completely randomized design (Systat version 19.0; SPSS, Shanghai). Subsequently, Students-Newman-Keuls test was conducted for multiple comparisons. *P*<0.05 was considered to be statistically significant.

## Results

### Behavioral test

**Figure 1:**
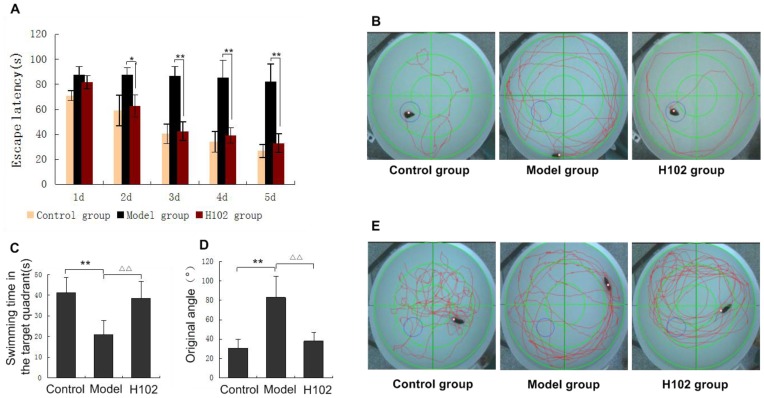
Paths and performance of mice in the probe trial. **A**: Escape latency in the three groups from day 1 to 5. (Day 2: *P<0.05; Days 3, 4, 5: **P<0.01). **B**: The photographs, from the image capture system of Morris water maze, reflected the escape-latency of APP mice. **C**: Swimming time in the target quadrant. (**P<0.01, ^▵▵^ *P*<0.01) **D**: Original angle. (**P<0.01, ^▵▵^ *P*<0.01) **E**: The photographs, from the image capture system of Morris water maze, recorded spatial probe process of APP mice. Error bars represent mean ± SEM. Statistical analysis was performed using repeated measures ANOVA and Student-Newman-Keuls test.

We tested mice with Morris water maze for behavioral analysis. The experiment lasted six days. Escape latency was tested in the first five days of training, and the so-called spatial probe test was conducted in the last day of training without platform. During the first 5 days, no significant difference was found between the control group and the H102 group. The difference was significant between the H102 group and the model group from day 2 to day 5. The result of escape latency as shown in Fig. 1A and 1B, indicated that the time for finding the platform by both the H102 group and the control group was descending with the increasing time of training. The result of the spatial probe test is shown in Fig. 1C–1E. The platform was removed on the sixth day. The staying time in the third quadrant (Fig. 1C) and original angle (Fig. 1D) were used as the indexes to estimate their memory. There was significant difference in swimming time in the target quadrant between the model group and the control group (*P*<0.01), which suggested that the model group had a lower ability to find platform than the control group. The difference between the H102 group and the model group was significant (*P*<0.01). No significant difference was seen between the H102 group and the control group. Mice in the model group had a lower ability to find the platform (*P*<0.01). Nevertheless, H102 group had a much higher ability to find the platform than the model group (*P*<0.01). There was no significant difference between the control group and the H102 group. Compared with the model group, the number of times the H102 group crossed the platform was significantly increased,as shown in Fig. 1E.

### Influence of H102 on the expression of inflammatory factors (iNOS, IL-1β and TNF-α)

**Figure 2:**
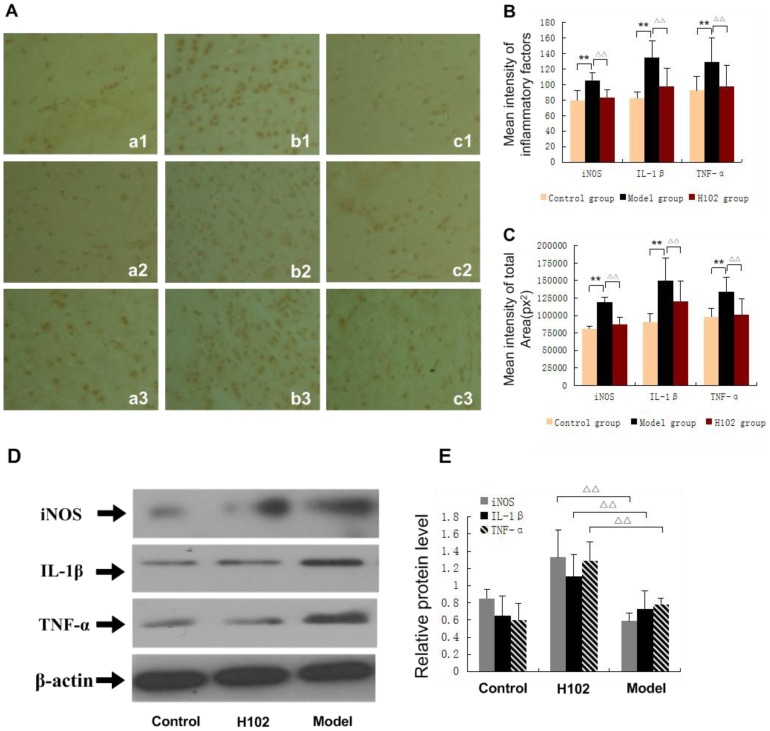
Expression of inflammatory factors (iNOS, IL-1β and TNF-α) in hippocampal CA1 area. **a**: control group; **b**: model group; **c**: H102 group. **1**: iNOS; **2**: IL-1β; **3**: TNF-α, (magnification ×400). **A**: The expression of inflammatory factors stained by IHC. **B**, **C**: Mean intensity of inflammatory factors and positive area of inflammatory factors. (**P<0.01, ^▵▵^ *P*<0.01) **D**, **E**: The expression of iNOS, IL-1β and TNF-α by Western blotting (^▵▵^ *P*<0.01). Error bars represent mean ± SEM. Statistical analysis was performed using one-way ANOVA following Student-Newman-Keuls test.

Expression of inflammatory factors presented as globular texture under microscope mostly happened in the cytoplasm at the areas of hippocampus CA1. Compared with control group and H102 group, three positive cells for the expressions of iNOS, IL-IL-1β and TNF-α were more distinctly stained and widely distributed in the model group (*P*<0.01), there was no significant difference between the control group and H102 group. As shown in Fig. 2D and 2E, compared with the model group, the expression of iNOS, IL-1β and TNF-α in H102 group presented a significant decrease, and there was no significant difference between control group and H102 group.

### Influence of H102 on the expression of P-tau, GSK-3 and PP-2A

**Figure 3:**
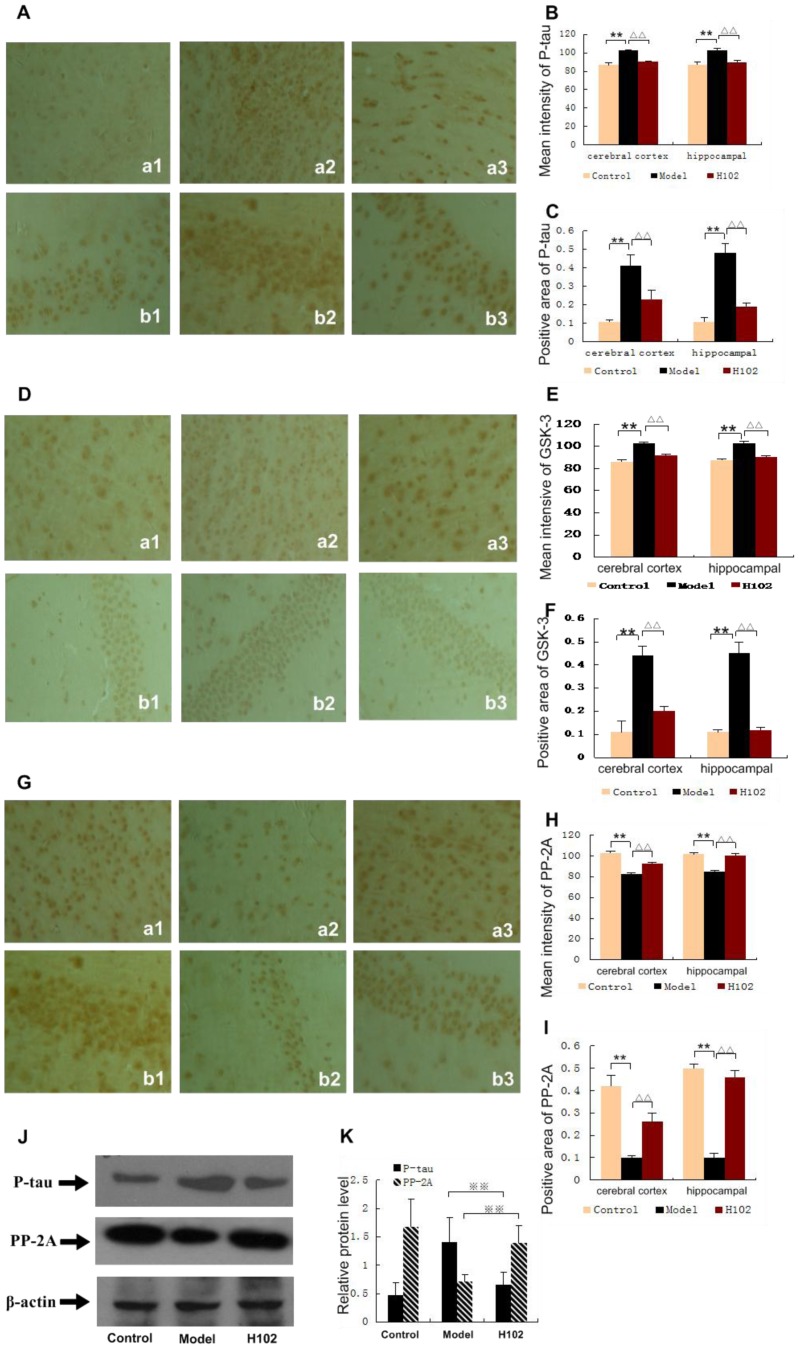
Expression of P-tau, GSK-3 and PP-2A in cerebral cortex and hippocampal area. a: cerebral cortex; b: hippocampal area. 1: control group; 2: model group; 3: H102 group, (magnification ×400). **A**: Expression of P-tau stained by IHC. **B**, **C**: Mean intensity of P-tau and positive area of P-tau. Content of P-tau (^**^ *P*<0.01, ^▵▵^ *P*<0.01) **D**: Expression of GSK-3 stained by IHC. **E, F**: Mean intensity of GSK-3 and positive area of GSK-3. (**P<0.01). **G:** Expression of PP-2A stained by IHC. **H, I:** Mean intensity of PP-2A and positive area of PP-2A. (**P<0.01). **J, K:** Expression of P-tau and expression of PP-2A by western blotting (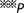
<0.01, 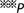
<0.01). Error bars represent mean ± SEM. Statistical analysis was performed using one-way ANOVA following Student-Newman-Keuls test.

The immunohistochemical test showed that there were much more cells which expressed P-tau and GSK-3 in the brain of the mice in the model group than in the control group (*P*<0.01), but compared with the model group, there were fewer cells expressing P-tau and GSK-3 in H102 group (*P*<0.01). However, more cells expressed PP-2A in the brain of the mice in H102 group, but fewer cells expressed PP-2A in the model group (*P*<0.01). The Western blotting showed that the content of P-tau in the brain of the model group was higher than that of the control group (*P*<0.01), but the content of PP-2A was lower (*P*<0.01). The protein expression level of P-tau in the brain of the mice of H102 group was lower, but the level of the PP-2A was higher, compared with the model group (*P*<0.01),(Fig. 3).

### Effect of H102 on cell apoptosis in the APP transgenic mouse brain

**Figure 4:**
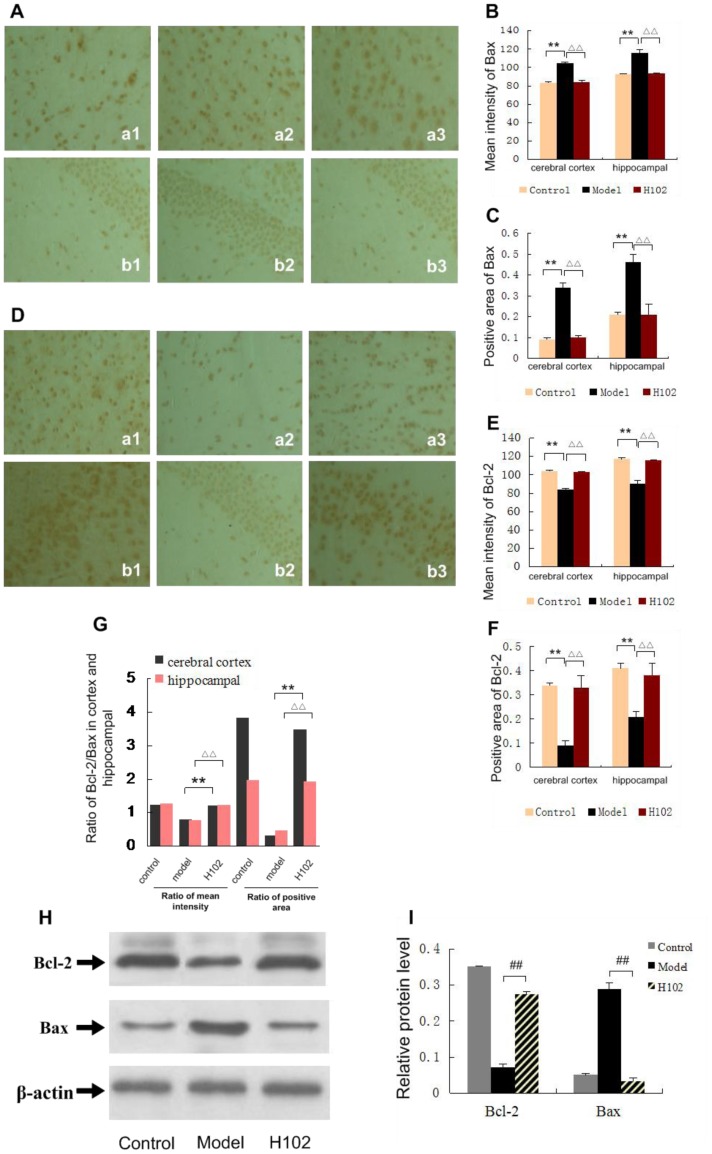
Expression of Bcl-2 and Bax in cerebral cortex and hippocampal area. a: cerebral cortex; b: hippocampal. 1: control group; 2: model group; 3: H102 group (magnification ×400). **A**: Expression of Bax stained by IHC. **B**, **C**: Mean intensity of Bax and positive area of Bax. (**P<0.01, ^▵▵^ *P*<0.01). **D**: Expression of Bcl-2 stained by IHC. **E, F**: Mean intensity of Bcl-2 and positive area of Bcl-2 (**P<0.01). **G**: The ratio of Bcl-2/Bax (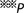
<0.01). **H, I**: Expression of Bcl-2 and Bax by Western blotting (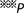
<0.01). Error bars represent mean ± SEM. Statistical analysis was performed using one-way ANOVA following Student-Newman-Keuls test.

By Bax immunohistochemical staining, the positive cells in the cortex and hippocampus CA3 area were decreased in H102 group compared with model group (*P*<0.01), and there was no significant difference between control group and H102 group. In Bcl-2 immunohistochemical staining, positive cells in the cortex and hippocampus CA3 area were increased in H102 group (*P*<0.01) compared with model group, and there was no significant difference between control group and H102 group. As shown in Fig. 4G, compared with model group, the ratio of Bcl-2/Bax was obviously increased in H102 group (*P*<0.01), and there was no significant difference between control group and H102 group.

Western blotting showed that the expression of Bax was obviously decreased in H102 group compared with model group (*P*<0.01), and there was no significant difference between control group and H102 group. Compared with model group, the expression of Bcl-2 was significantly enhanced in H102 group (*P*<0.01), and there was no significant difference between control group and H102 group (Fig. 4).

## Discussion

According to the amyloid hypothesis, aggregation of Aβ in the brain plays a primary role in the pathogenesis of AD [9]. Aggregation of Aβ is preceded by the formation of nonfibrillar species (protofibrils), which are in turn preceded by oligomeric species [28]. Recent evidences demonstrate that soluble oligomers of Aβ may be responsible for synaptic dysfunction in the brains of AD patients [9]. Moreover, their pathogenic relevance is supported by the finding that oligomer formation increases with the progression of AD, resulting in mutations in APP [29].

As is known, abnormalities are thought to play an important role in the pathologic processes of AD via influences on the synthesis and degradation of A-beta and AD is also characterized by the deposition of aggregates of the β-amyloid peptide (Aβ) in the brain. While, the synthetic of beta-sheet structure can be a potential therapeutic strategy for AD, as it is capable of binding A-beta but unable to become part of a beta-sheet structure, thus inhibiting the peptide aggregation [30]. β-sheet breaker peptides are specifically synthesized inhibitors against Aβ aggregation, which have been developed as drugs for the treatment of AD. A series of β-sheet breaker peptides were designed by Soto et al. [20]; iAβ11 and iAβ5 have been proved to be effective in preventing β-sheet formation and inhibiting Aβ aggregation both *in vitro* and *in vivo*. Many studies suggested that membranes played a key role in the Aβ aggregation, so researches about the interactions between the β-sheet breaker peptide acetyl-LPFFD-amide, iAβ5p and the Aβ(25-35) fragment with lipid membranes were significant [31]. Some researchs showed that iAβ5p influenced the Aβ(25-35) interaction with the bilayer through a cholesterol-mediated mechanism: iAβ5p withheld cholesterol in the inner hydrophobic core of the bilayer, making the interfacial region more fluid and capable to accommodate Aβ(25-35). As a consequence, iAβ5p prevented the Aβ(25-35) release from the lipid membrane, which was the first step of the β-amyloid aggregation process [32].

The aim of this study was to confirm the β-amyloid hypothesis via analyzing the pharmacological properties of a 10-residue β-sheet breaker peptide H102 active in reducing amyloid load and cerebral damage in animal models of AD in an attempt to provide a firmly theoretical basis for the feasibility of H102 in the treatment of AD. β-sheet breaker peptides are short synthetic peptides homologous to the central fragment of Aβ containing residues that destabilize β-sheet structures. Based on the principle of Aβ aggregation and the biochemical structure of Aβ, we had designed a series of β-sheet breaker peptides using the Drug Design Studio-VM system in our previous work. To combine the designed objects with hydrophobic sequences which are now widely recognized as the basic pathological link of Aβ aggregation [33], we have designed these polypeptide materials targeting Aβ with a relatively high solubility and activity using the three-dimensional display technology in order to pathologically block the progression of AD. These polypeptides directly interact with Aβ to inhibit amyloid-mediated synaptic dysfunction, oligomer and Aβ fibrillogenesis followed by amyloid formation and neurotoxicity. We assayed and compared the pharmaceutical effects of six polypeptide inhibitors on Aβ aggregation and protofibril formation *in vitro* by fluorescence analysis and transmission electron microscopy in order to select the optimal one. We found that all of them, particularly H102, were able to bind to Aβ1-42 and inhibit Aβ fibrillogenesis and neurotoxicity in a concentration-dependent manner, in addition to the effects of neuroprotection and neurotrophy on neurons [34].

Based on the previous studies, using the APP transgenic mouse models, the expression of APP, Aβ and senile plaque in brains was examined for observing the pharmacological mechanism of H102. The results indicated that H102 did have the ability to reduce Aβ, which provided important experimental evidence in the research and development of new drugs for treatment of AD. Degrading obstacle is one of the causes of Aβ aggregation. Depressed expression of insulin-degrading enzyme (IDE) and neprilysin (NEP) seems to induce Aβ formation, and up-regulation of IDE and NEP might lower the level of Aβ25. Our results show that H102 can increase the activity of NEP and IDE [24]. The expression of synapse-associated proteins (synaptophysin, PSD-95 and Shank-1) was lowered in the model group [25], indicating the damage to the hippocampal synaptic function by Aβ aggregation. Nevertheless, expressions of the three proteins were increased in H102 groups when compared with the model, which implicated that H102 may improve the synaptic function and plasticity in brains of APP transgenic mice.

Neuroinflammation is one of the key characteristics of AD, and the inflammatory process is mediated by the microglia (MG), which participated in the immune response. MG, which is a central nervous system immune cell, plays a similar role in the brain macrophage cell function, and in the action against infection, MG is activated into reactive MG, which secretes inflammatory factors, such as TNF-α, IL-1 and TGF-β. This process results in a continuous increase of brain cell factor levels of AD patients and toxic effects of neurons. A recent study confirmed that Aβ protein can activate MG directly to release inflammatory mediators, such as interleukin-1β (IL-1β) and tumor necrosis factor-α (TNF-α), and then to produce cell factors and neural toxic substances. Some inflammatory mediators in turn induce MG chemotaxis and activation, and others lead to local tissue damage and damage of neurons [35]. Although the above mechanisms remained unclear, the interaction between Aβ and MG cell surface receptors has been verified, and MG activation is found in both AD patients and AD transgenic animal models [36]. Activated MG releases inflammatory factors, leading to obvious expression of neuron, APP, and the formation of neuron fiber tangles [37], [38]. These inflammatory factors play an important role in the pathological progress of AD. A large number of researches have confirmed that the inflammatory factors can form and develop P-tau lesions in AD [39]. And there is a close relationship between expression level of inflammatory factors in AD brain and tau protein lesions. P-tau is a kind of phosphorus protein, and the degree of phosphorylation can adjust the biological activities. In patients with AD, abnormal phosphorylation of P-tau in the brain is associated with the regulatory dysfunction of glycogen synthase kinase-3(GSK-3), and protein phosphatase (PP) [40]. GSK-3 includes the alpha and beta enzymes, but GSK-3β plays a key role in phosphorylation of P-tau. And protein phosphatase PP-2A and PP-1 *in vitro* can make P-tau dephosphorylate in different positions [29]. PP-1 can adjust activity of protein kinase GSK-3. Normal activity of phosphatase maintains the integrity of the cytoskeleton, and reduced activity of phosphatase will lead to excessive phosphorylation of P-tau. So inhibiting PP-2A activity may cause abnormal phosphorylation of P-tau. Therefore, PP-1 and PP-2A will be the target for the treatment of AD. In our experiment, through reversing the production of Aβ to reduce the content of the P-tau and improve the expression of the PP-2A and PP-1, H102 not only reduced the damage of the neurons but also promoted the reconstruction of the nervous system, thus improving the ability of learning and memory of the APP transgenic mice.

Numerous studies have demonstrated that Aβ is a predominant factor in the process of AD, and the cell apoptosis plays an important role in the pathogenesis of AD. We chose the meaningful apoptosis proteins as a research index, including Bax protein and Bcl-2 protein. These proteins played a key role in the process of cell apoptosis, and the content of the protein directly decides the cell survival or apoptosis [41]. Bax protein is an apoptosis-induced protein, and Bcl-2 protein is an apoptosis-inhibiting protein, which can prevent cell apoptosis. The ratio of Bcl-2 and Bax determines the cell apoptosis, the higher ratio the less cell apoptosis, vice versa. We have found that H102 can reduce the expression of Bax, but increase the expression of Bcl-2.

Morris water maze, which measures the space learning and memory capacity, is used to assess the influence of the factors, such as AD, on the learning and memory of rodents. The results of Morris water maze in cognitive ability of APP transgenic mice showed that the escape latency in APP mice was much shorter as compared with H102-pretreated group, which suggested that H102 was effective in improving the learning and information acquiring abilities. Moreover, target quadrant retention time was much longer in APP group as compared with the H102-pretreated group, which indicated that H102 can improve the abilities of learning and memory of mice.

Much evidence indicates that the memory and cognitive deficits of patients with AD are closely associated with dysfunction of central cholinergic system. The degree of reduced choline acetyltransferase (ChAT) activity in cerebral cholinergic neurons is significantly correlated with the severity of dementia or cognitive impairment observed in AD [42]. In about half of the AD cases, cortical choline actyltransferase (ChAT) activity was markedly reduced in spite of preserved nucleus basalis of Meynert ChAT activity, suggesting a deficiency of cortical origin and/or of axonal transport in AD [43]. Acetylcholinesterase (AChE) modulates acetylcholine to proper levels by degradation, thus, excessive AChE activity leads to constant acetylcholine deficiency, causing memory and cognitive impairment in AD [26]. AChE was found to increase amyloid fibril assembly with the formation of highly toxic complexes (Abeta-AChE) and the neurotoxic effect induced by Abeta-AChE complexes both *in vitro* and *in vivo* [44]. The density of AChE-rich fibers mainly located in cerebral cortex plays an important role in cholinergic neurotransmission, and a dramatic loss was found in the brains of patients with AD [45]. Our previous researches demonstrated that H102 was able to obviously improve the activities of ChAT, and lower the activity of AChE [27]. Aβ resulted in free radical damage by increasing the H_2_O_2_ levels and exhibited cytotoxic action on neurons, which could be reversed by anti-oxidants. Aβ-induced oxidative stress leads to neurodegeneration in AD brain, which can be inhibited by free-radical antioxidants [46]. Superoxide dismutase (SOD), an endogenous antioxidant enzyme, plays an important role in the intracellular antioxidant defense in the brain. We measured the SOD activity in APP transgenic mice and found that the AD model mice had remarkably reduced activity of SOD and treatment with H102 significantly increased the SOD activity. These data indicated that H102 could improve the activity of SOD, which involves the clearance of free radicals, and protect the brain from oxidative stress [27]. Malondialdehyde was considered as an index of lipid peroxidation [47]. Our results showed that the level of malondialdehyde (MDA) was markedly higher in model group than in the control group, which is in agreement with the previous study. H102-treated group presented a significant reduction in the level of MDA compared with model group [27]. Therefore, H102 might prevent lipid peroxidation from producing MDA and alleviate the toxicity of MDA on the brain of APP695 transgenic mice.

We will focus on pharmacokinetic studies to analyze the metabolic characteristics and blood-brain barrier (BBB) passing rate of H102 via experimental evaluation *in vitro*. Peptides are amenable to the process of rationale drug development. It is relatively easy to develop peptides with an *in vitro* activity, a high selectivity, and a low toxicity. However, a major drawback with the use of peptides as drugs for neurological diseases is their rapid metabolism by proteolytic enzymes and their poor BBB permeability [20], [48]. Due to their predisposition for enzymatic degradation, unmodified peptides do not circulate in blood for more than a few minutes. Moreover, they have a generally poor bioavailability in tissues and organs, thus limiting their usefulness as therapeutic agents. Therefore, when used as a drug, the peptides have to be chemically modified so as to diminish the proteolytic degradation and enhance the bioavailability. The advance in the field of peptide solid- and liquid-phase synthesis allows the introduction of a wide variety of chemical modifications into a peptide backbone [22], [49]. These modifications are useful only if they are strategically introduced to maximize enzymatic stability and bioavailability and simultaneously preserve or enhance the potency and selectivity of the bioactive peptide. This approach requires knowledge of the major *in vivo* proteolytic sites of the peptide and the chemical groups in the molecule responsible for its biological activity [22].

In conclusion, the present study shows that H102 has a protective effect on APP695 transgenic mice which have been documented as a valuable model in the study of AD. Therefore, H102, as a β-sheet blocker peptide, shows great potential for the treatment of AD.
